# 718. Sulbactam Susceptibility Testing by Gradient Diffusion Method (E-test) in Carbapenem-Resistant *Acinetobacter baumannii* (CRAB) from a Tertiary Care Hospital in Thailand

**DOI:** 10.1093/ofid/ofad500.780

**Published:** 2023-11-27

**Authors:** Chris Fujitnirun, Sariya Dungdonbom, Pimpha Rungnobhakhun

**Affiliations:** Bhumibol Adulyadej Hospital, Bangkok, Krung Thep, Thailand; Bhumibol Adulyadej Hospital, Bangkok, Krung Thep, Thailand; Bhumibol Adulyadej Hospital, Bangkok, Krung Thep, Thailand

## Abstract

**Background:**

Introduction: CRAB is one of the major pathogens of nosocomial infection in Thailand and also cause a great burden to global public heath. Current treatments for CRAB are limited because of multidrug resistance and drug toxicity especially renal toxicity associated with colistin. ESCMID guidelines and IDSA guidance in 2021 recommended sulbactam as the first line treatment for sulbactam susceptible CRAB. Here we reported susceptibility data of CRAB to sulbactam in a tertiary care hospital in Thailand.

**Methods:**

CRAB were isolated from from inpatients admitted to Bhumibol Adulyadej Hospital, a 600-bed tertiary government hospital in Bangkok, Thailand, from February to April 2023. The minimal inhibitory concentration (MIC) of sulbactam was determined by the gradient diffusion method (E-test strip, Qilu Antibiotics) using Mueller-Hinton agar plates and reported by an experienced clinical microbiologist.

**Results:**

Among 27 isolates of CRAB, sputum is the most common source of the organism (70%) (figure 1). The MIC range of sulbactam was 8 to ≥ 256 μg/ml. Median MIC was 32 μg/ml but the most frequently reported MIC was ≥ 256 μg/ml (41%) and 89% of the isolates showed MIC ≥ 24 μg/ml as shown in figure 2.

Figure 1

**Figure:**
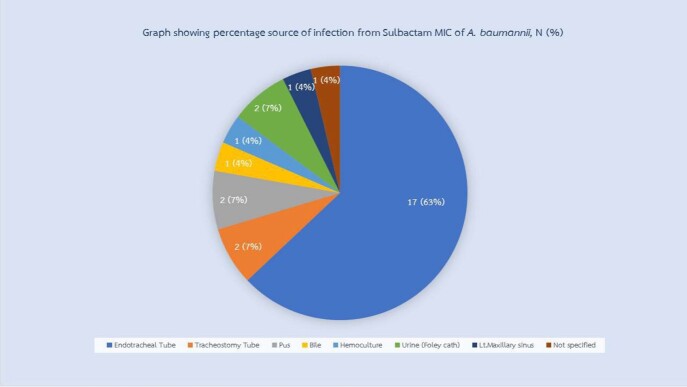

Source of A. baumannii isolates

Figure 2

**Figure:**
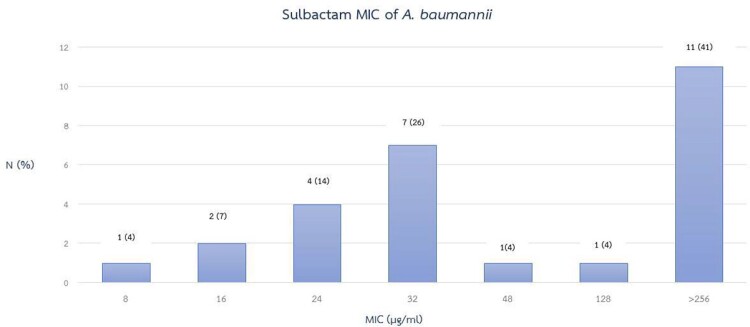

MIC of A. baumannii isolates

**Conclusion:**

At the present time, there was only breakpoint for sulbactam in combination with ampicillin. Clinical breakpoint of sulbactam alone for *A. baumannii* was not defined by current documents of both CLSI and EUCAST. Current recommendations which suggest 9 g/day of sulbactam for CRAB infection based on data from “less susceptible” CRAB (sulbactam MIC > 4 but ≤16 μg/ml). Our study reported isolates of CRAB that showed very high sulbactam MIC. These findings caution that local sulbactam susceptibility pattern should be defined before using sulbactam as the first line empirical treatment for CRAB infection.

**Disclosures:**

**All Authors**: No reported disclosures

